# Predictive Value of Serum Pepsinogen and Gastrin‐17 in Patients With Acute Coronary Syndrome for Post‐Percutaneous Coronary Intervention Oral Dual Antiplatelet Therapy‐Associated Upper Gastrointestinal Bleeding

**DOI:** 10.1155/cdr/8095418

**Published:** 2026-07-13

**Authors:** WanPing Yao, Chang Liu, ZiYi Fang, ShanShan Li, Lu Hong Xu, WenHao Qian, Fangfang Li, Jing Zong

**Affiliations:** ^1^ Department of Cardiology, The Affiliated Hospital of Xuzhou Medical University, Xuzhou, Jiangsu, China, xzmc.edu.cn; ^2^ Institute of Cardiovascular Disease, Xuzhou Medical University, Xuzhou, Jiangsu, China, xzmc.edu.cn

**Keywords:** dual antiplatelet therapy (DAPT), gastrin-17, P_2_Y_12_ receptor antagonist, precise-DAPT scores, proton pump inhibitors(PPIs), upper gastrointestinal bleeding(UGIB)

## Abstract

**Background:**

Gastrin‐17 (G‐17) and serum pepsinogen (PG) are indicators that reflect the structure and function of the stomach mucosa. Although they have been linked to upper gastrointestinal bleeding (UGIB) in peptic ulcer disease, it is unknown how they relate to the risk of UGIB in patients with acute coronary syndrome (ACS) after dual antiplatelet treatment (DAPT) following percutaneous coronary intervention (PCI).

**Objective:**

This single‐center, retrospective study aims to evaluate whether PG and G‐17 as gastric mucosal assessment markers, are potentially associated with UGIB during DAPT following PCI in patients with ACS.

**Methods:**

This study employed a retrospective analysis design and included 334 patients with ACS (2021–2023) from Xuzhou Medical University Affiliated Hospital, divided into UGIB group (69 patients) and non‐UGIB group (265 patients). Clinical and laboratory data were collected. Multivariate logistic regression and ROC curve analysis were used to evaluate the associations of G‐17, PRECISE‐DAPT scores, ACS subtype, and P_2_Y_12_ receptor antagonist used for UGIB after DAPT.

**Results:**

Compared with the non‐UGIB group, G‐17 levels and PRECISE‐DAPT scores in the UGIB group were markedly higher, and ticagrelor was prescribed more commonly (*p* < 0.001). The area under the curve (AUC) of G‐17, PRECISE‐DAPT scores alone, and in combination were 0.734, 0.794, and 0.844, respectively; the AUC further increased to 0.878 after incorporating ticagrelor exposure, indicating that the combined assessment with ticagrelor exposure has superior exploratory value in identifying high‐risk patients with UGIB (*p* < 0.05). Furthermore, the combined use of PPIs during DAPT reduced the incidence of UGIB (34.78% vs. 65.22%, *p* < 0.05), which serves as an important interfering factor for G‐17 assessment.

**Conclusion:**

Higher G‐17 levels and PRECISE‐DAPT scores were associated with UGIB during DAPT after PCI. Combined assessment with ticagrelor exposure showed exploratory value for identifying patients at higher bleeding risk, although PPIs use influenced model performance. Given the retrospective single‐center design and limited sample size, these findings remain preliminary and require prospective multicenter validation.

## 1. Introduction

ACS is primarily categorized into three types: unstable angina (UA), non‐ST‐segment elevation myocardial infarction (NSTEMI), and ST‐segment elevation myocardial infarction (STEMI). In recent years, the incidence of ACS has been rising, posing a serious threat to human health and life safety [1]. PCI has become the primary revascularization modality for patients with ACS and patients with chronic coronary syndrome (CCS) [2, 3], while DAPT:(aspirin + P_2_Y_12_ Receptor Antagonist) serves as the cornerstone therapy for preventing stent thrombosis and reducing myocardial infarction (MI), stroke, and cardiovascular mortality following PCI [4]. However, bleeding (particularly UGIB) represents the primary safety concern associated with DAPT. Research indicates that the incidence of bleeding events has risen significantly with the widespread use of antiplatelet agents, with UGIB being the most common type, accounting for 48.7% of all bleeding events. Furthermore, bleeding events are closely associated with poor outcomes, such as increased mortality and prolonged hospital stays [5, 6].

Relevant studies report that in patients undergoing drug‐eluting stent implantation, extending DAPT to 30 months versus 12 months significantly reduced the risk of stent thrombosis (0.4% vs. 1.4%) and major adverse cardiovascular and cerebrovascular events (4.3% vs. 5.9%), but also markedly increased the risk of moderate to severe bleeding (2.5% vs. 1.6%) [7]. This underscores the critical importance of accurately identifying high‐risk bleeding populations when balancing the benefits and risks of DAPT.

Furthermore, the ESC guidelines explicitly recommend PPIs for DAPT patients with a grade IA recommendation (regardless of bleeding risk). In contrast, the ACC/AHA guidelines only recommend PPIs (grade IA) for those with a history of gastrointestinal bleeding [8]. This indicates that guidelines are necessary for gastric mucosal protection, yet there remains a lack of individualized recommendations for populations with impaired gastric function. Current traditional risk factors (such as age and renal function), and the PRECISE‐DAPT score proposed by the ESC in 2017 [9] to predict bleeding risk in patients undergoing DAPT after PCI, fail to cover gastrointestinal‐specific risks adequately. Consequently, it remains challenging to provide clinically tailored medication guidance for high‐risk individuals with impaired gastric function. This study aims to explore whether PG and G‐17 as gastric mucosal assessment markers are potentially associated with UGIB during DAPT following PCI in patients with ACS. It seeks to assist clinicians in early identification of gastric mucosal status and high‐risk populations for UGIB, enabling the formulation of personalized treatment strategies.

## 2. Methods

### 2.1. Study Design and Participants

This retrospective analysis included medical records of patients diagnosed with ACS at Xuzhou Medical University Affiliated Hospital between January 1, 2021 and July 31, 2023 who underwent PCI within 24 h and received postoperative therapy with aspirin plus a P_2_Y_12_ Receptor Antagonist (clopidogrel or ticagrelor). Ultimately, 334 patients with ACS were selected and enrolled. The study was conducted in accordance with the Helsinki Declaration and approved by the Ethics Committee of the Affiliated Hospital of Xuzhou Medical University (No: XYFY2025‐KL615‐01).

The following are inclusion criteria: ① All enrolled patients met the diagnostic criteria outlined in the 2021 ACC/AHA/SCAI Guidelines for Coronary Revascularization and underwent PCI with coronary stent implantation for revascularization following hospital admission [3]; ② All patients received DAPT (aspirin plus P_2_Y_12_ Receptor Antagonist) therapy postoperatively for a duration exceeding 6 months; ③ Diagnostic criteria for UGIB: Symptoms primarily manifested as hematemesis and melena, with or without concomitant palpitations, dizziness, or hypotension [10].

Exclusion criteria are as follows: ① Age < 18 years; ② History of PCI or coronary artery bypass grafting (CABG); ③ Concurrent gastrointestinal tumors, liver cirrhosis, or other gastrointestinal diseases such as gastric fundus varices; ④ Presence of malignancy, hematological disorders, immunological diseases, infectious diseases, severe hepatic or renal insufficiency, or recent major surgery; ⑤ Patients lost to follow‐up or with incomplete medical records.

### 2.2. General Information

Demographic and clinical data encompass patients’ gender, age, body mass index, medical history (such as hypertension, diabetes, smoking, and alcohol consumption history, etc.), discharge diagnosis, PPIs used, HP positive, and DAPT regimen at admission.

### 2.3. Laboratory Measurements

Fasting venous blood samples were collected within 24 h of admission and sent to the Laboratory Department of Xuzhou Medical University Affiliated Hospital for hematological testing, including complete blood count, biochemical analysis (covering liver function, renal function and eGFR, blood glucose, lipid profile), coagulation parameters, and gastric function indicators (PGI, PGII, and G‐17).

### 2.4. Follow‐Up and Outcome Events

This study conducted a retrospective follow‐up of all patients, with the follow‐up period ranging from the day of discharge to 2 years post‐discharge. The endpoints of the follow‐up were the first occurrence of UGIB during DAPT within 2 years, loss to follow‐up, or the study’s cut‐off date. Follow‐up was primarily achieved through telephone contact or by reviewing patients’ medical records. The specific timing of all UGIB events was recorded (number of days/months post‐PCI). Based on the time of the first UGIB occurrence, these were classified as early bleeding (within 30 days post‐PCI) and late bleeding (30 days post‐PCI to the end of follow‐up).

### 2.5. PRECISE‐DAPT Score

The PRECISE‐DAPT scoring system is calculated according to the 2017 ESC guidelines for predicting bleeding complications following stent implantation and subsequent dual antiplatelet therapy. It is based on the patient’s age, creatinine clearance, hemoglobin, white blood cell count, and history of spontaneous bleeding: a decrease of 15 mL/min in creatinine clearance adds 1 point; a reduction of 10 g/L in hemoglobin adds 1 point; an increase of 5 × 10^9^/L in white blood cell count adds 1 point; a history of spontaneous bleeding adds 10 points. The total score ranges from 0 to 100 points. A score < 25 indicates low bleeding risk, with a recommendation to extend the duration of DAPT; a score ≥ 25 indicates high bleeding risk, with a recommendation to shorten the duration of DAPT [9].

### 2.6. Statistical Analysis

All statistical analyses and graphical representations in this study were conducted using SPSS Version 26.0 and R Version 4.2. Patients were categorized into two groups: UGIB group (*n* = 69) and non‐UGIB group (*n* = 265). Quantitative data were first subjected to Shapiro–Wilk normality testing. For data that followed a normal distribution, independent‐samples *t*‐tests were used for statistical analysis, and results were expressed as mean ± standard deviation (x ± s). For data that did not follow a normal distribution, Mann–Whitney *U* tests were employed, with results expressed as median [M (Q1, Q3)]. Qualitative data were presented as case numbers and percentages (%), and intergroup comparisons utilized *χ* ^2^ tests. Univariate and multivariate logistic regression analyses were conducted to assess factors influencing UGIB occurrence in patients receiving DAPT after PCI. ROC curves were conducted to assess the association between G‐17, the PRECISE‐DAPT scores, the P_2_Y_12_ receptor antagonist, and combinations of these indicators with the incidence of UGIB among patients undergoing DAPT after PCI. The Youden index was employed to determine optimal cut‐off values. Because of the impact of PPIs use on G‐17 levels, the DeLong non‐parametric test was employed to compare the effects of PPIs use on the performance of G‐17. A two‐tailed *p* value of less than 0.05 was considered statistically significant for all analyses.

## 3. Results

### 3.1. Baseline Characteristics of the Study Participants

This study enrolled 334 patients, including 265 (79.3%) in the non‐UGIB group and 69 (20.7%) in the UGIB group. No significant intergroup differences were found in pepsinogen levels and other demographic characteristics (*p* > 0.05; Table 1). By contrast, significant differences existed in age, G‐17 level, PRECISE‐DAPT score, ACS subtype (UA, NSTEMI, STEMI), P_2_Y_12_ receptor antagonist type (clopidogrel, ticagrelor), PPI use, and Hp positivity (*p* < 0.05; Table 1).

**Table 1:** Baseline characteristics of the study participants.

Variable	Non‐UGIB group (*n* = 265)	UGIB group (*n* = 69)	t/Z/*χ* ^2^	*p*
Age (years)	64.00 (56.00, 71.00)	71.00 (67.00, 77.00)	*Z* = −4.98	< 0.001
BMI (kg/m^2^)	24.49 (22.49, 26.83)	24.22 (22.67, 27.55)	*Z* = −0.33	0.739
Sex (n%)			*χ* ^2^ = 1.71	0.191
Female	165 (62.26)	37 (53.62)		
Male	100 (37.74)	32 (46.38)		
Hypertension (*n* %)			*χ* ^2^ = 0.05	0.818
No	100 (37.74)	25 (36.23)		
Yes	165 (62.26)	44 (63.77)		
Diabetes (*n* %)			*χ* ^2^ = 4.23	0.040
No	173 (65.28)	54 (78.26)		
Yes	92 (34.72)	15 (21.74)		
Smoking history (*n* %)			*χ* ^2^ = 0.37	0.543
No	194 (73.21)	53 (76.81)		
Yes	71 (26.79)	16 (23.19)		
Alcohol consumption (*n* %)			*χ* ^2^ = 3.35	0.067
No	218 (82.26)	63 (91.30)		
Yes	47 (17.74)	6 (8.70)		
WBC (10^9^/L)	6.00 (5.00, 7.30)	6.40 (5.00, 8.00)	*Z* = −0.59	0.556
HB (g/L)	138.00 (128.00, 148.00)	136.00 (118.00, 147.00)	*Z* = −1.76	0.078
PLT (10^9^/L)	208.00 (178.00, 253.00)	213.00 (175.00, 252.00)	*Z* = −0.38	0.707
TBIL (*μ*mol/L)	10.80 (8.40, 15.00)	11.70 (7.50, 14.40)	*Z* = −0.23	0.815
AST (U/L)	21.10 ± 10.66	21.19 ± 11.80	*t* = −0.06	0.951
ALT (U/L)	22.09 ± 15.67	19.35 ± 14.80	*t* = 1.31	0.192
TBA (*μ*mol/L)	4.03 ± 3.14	4.86 ± 2.91	*t* = −1.97	0.049
BUN (mmol/L)	5.20 ± 1.28	5.53 ± 1.79	*t* = −1.41	0.161
CREA (*μ*mol/L)	60.64 ± 13.46	64.29 ± 17.45	*t* = −1.62	0.109
Cys‐C (mg/L)	0.89 ± 0.16	0.99 ± 0.23	*t* = −3.80	< 0.001
GFR (mL/min)	111.00 ± 13.16	103.34 ± 17.11	*t* = 3.46	< 0.001
HbA1c (%)	6.26 ± 1.08	5.99 ± 0.72	*t* = 1.97	0.050
TG (mmol/L)	1.78 ± 1.84	1.62 ± 0.83	*t* = 0.73	0.467
PGI (*μ*g/L)	183.12 ± 84.09	205.68 ± 113.19	*t* = −1.55	0.125
PGII (*μ*g/L)	18.59 ± 12.49	21.98 ± 14.23	*t* = −1.95	0.052
PGR	13.17 ± 8.74	13.22 ± 11.74	*t* = −0.04	0.970
G‐17 (pmol/L)	17.24 ± 19.39	34.60 ± 22.31	*t* = −5.91	< 0.001
PRECISE‐DAPT score	9.27 ± 5.29	16.75 ± 7.94	*t* = −7.41	< 0.001
ACS type (*n* %)			*χ* ^2^ = 20.30	< 0.001
UA	197 (74.34)	32 (46.38)		
NSTEMI	53 (20.00)	27 (39.13)		
STEMI	15 (5.66)	10 (14.49)		
P₂Y₁₂ Receptor Antagonist (*n* %)			*χ* ^2^ = 6.29	0.012
Clopidogrel	198 (74.72)	41 (59.42)		
Ticagrelor	67 (25.28)	28 (40.58)		
PPIs used (*n* %)			*χ* ^2^ = 8.38	0.004
No	121 (45.66)	45 (65.22)		
Yes	144 (54.34)	24 (34.78)		
HP positive (*n* %)			*χ* ^2^ = 7.08	0.008
No	141 (53.21)	49 (71.01)		
Yes	124 (46.79)	20 (28.99)		

*Note:* Data are presented as *m* *e* *a* *n* ± *S* *D*, median (Q1, Q3), or *n* (%).

Abbreviations: ALT, alanine aminotransferase; AST, aspartate aminotransferase; BMI, body mass index; BUN, blood urea nitrogen; CREA, creatinine; Cys‐C, cystatin‐C; G‐17, Gastrin 17; GFR, glomerular filtration rate; HB, hemoglobin; HbA1c, glycated hemoglobin; HP, Helicobacter pylori; NSTEMI, non‐ST‐segment elevation myocardial infarction; PGI, pepsinogens I; PGII, pepsinogens II; PGR, pepsinogen ratio; PLT, platelet; PPIs, proton pump inhibitors; STEMI, ST‐segment elevation myocardial infarction; TBA, total bile acid; TBIL, total bilirubin; TG, total triglyceride; UA, unstable angina; WBC, white blood cell.

### 3.2. Analysis of Factors Influencing UGIB by the Multivariate Logistic Regression

After adjusting for confounding factors such as age, sex, medical history, and HP positive, multivariate logistic regression analysis was conducted. The occurrence of UGIB was set as the dependent variable, and relevant risk factors were taken as independent variables. The results showed that higher G‐17 levels (OR = 1.098, 95% CI: 1.066–1.131, *p* < 0.001) and PRECISE‐DAPT scores (OR = 1.327, 95% CI: 1.174–1.501, *p* < 0.001), and ticagrelor application (OR = 15.697, 95% CI: 3.951–62.359, *p* < 0.001) were all associated with the high incidence of UGIB following DAPT (see Table 2). The addition of PPIs to DAPT can reduce the incidence of UGIB (*p* < 0.001), suggesting that PPIs may serve as a potential indicator for reducing the incidence of UGIB.

**Table 2:** Analysis of factors influencing UGIB by the multivariate logistic regression.

Variable	*β*	SE	Wald *χ* ^2^	*p*	OR	95% CI
Sex (female)	0.105	0.481	0.219	0.827	1.111	0.433~2.849
Age (years)	0.020	0.041	0.499	0.618	1.021	0.942~1.106
Hypertension (YES)	−0.110	0.435	‐0.253	0.800	0.896	0.382~2.100
Smoking history (YES)	0.614	0.538	1.141	0.254	1.847	0.644~5.299
Alcohol consumption (YES)	0.047	0.715	0.066	0.947	1.049	0.258~4.258
G‐17	0.093	0.015	6.174	< 0.001	1.098	1.066~1.131
PRECISE‐DAPT Score	0.283	0.063	4.517	< 0.001	1.327	1.174~1.501
P_2_Y_12_ Receptor Antagonist (Ticagrelor)	2.753	0.704	3.912	< 0.001	15.697	3.951~2.359
ACS Type (NSTEMI)	0.253	0.491	0.516	0.606	1.288	0.492~3.369
ACS Type (STEMI)	0.063	0.738	0.086	0.932	1.065	0.251~4.522
PPIs used (Yes)	−5.452	1.429	−3.817	< 0.001	0.004	0.000~0.070
HP positive (Yes)	1.316	1.224	1.075	0.282	3.729	0.339~1.054

*Note:* gastrin‐17, PRESCISE‐DAPT score, ACS subtype (assigned: UA“0”, NSTEMI“1”, STEMI“2”); P₂Y₁₂ Receptor Antagonist (assigned: clopidogrel “1”; ticagrelor “2”), PPIs used, HP positive as independent variables, with the occurrence of UGIB following DAPT after PCI (assigned: present “1”; no occurrence “0”) as the dependent variable. Other abbreviations were as in Table 1.

Abbreviations: *β*, multiple regression coefficient; CI, confidence interval; HP, Helicobacter pylori; OR, odds ratio; *p*, probability value; PPIs, proton pump inhibitors; SE, standard error; Wald *χ* ^2^, Wald chi‐square.

### 3.3. ROC Curve Analysis of Risk Factors for Predicting UGIB Following DAPT

**Figure 1:**
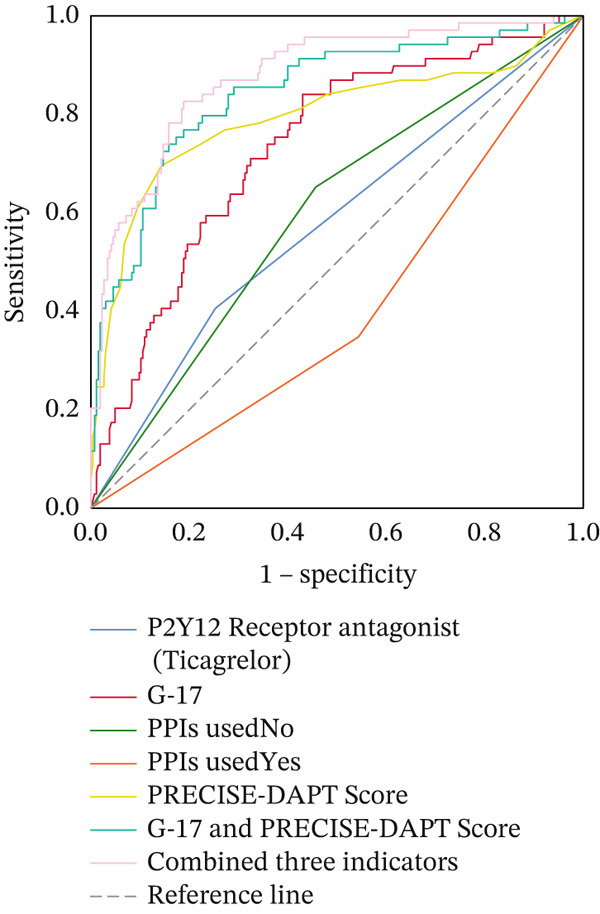
Receiver operating characteristic (ROC) curve of G‐17, PRECISE‐DAPT score, P_2_Y_12_ receptor antagonist use (ticagrelor), and their combined prediction of UGIB: Ticagrelor (*A* *U* *C* = 0.576, *p* = 0.050); G‐17 (*A* *U* *C* = 0.734, *p* < 0.001); PRECISE‐DAPT Score (*A* *U* *C* = 0.794, *p* < 0.001); PPIs used (Yes) (*A* *U* *C* = 0.402, *p* = 0.012); PPIs used (No) (*A* *U* *C* = 0.598, *p* = 0.012); Combine with G‐17 and PRECISE‐DAPT Score (*A* *U* *C* = 0.844, *p* < 0.001); Combine three indicators (*A* *U* *C* = 0.878, *p* < 0.001).

ROC curves were used to evaluate the potential exploratory value of G‐17, the PRECISE‐DAPT score, ticagrelor exposure, and PPIs use in the diagnosis of UGIB in patients undergoing DAPT after PCI. Results indicated that the AUC of G‐17 and PRECISE‐DAPT scores were 0.734 and 0.794, respectively. The combined assessment of the two markers showed an AUC of 0.844. Further combination with ticagrelor exposure increased the AUC to 0.878 (see Table 3 and Figure 1). This suggests that the combined assessment and ticagrelor exposure show exploratory value in identifying patients at higher risk of UGIB (*p* < 0.05). Furthermore, the use of PPIs is a confounding factor in evaluating UGIB. They may influence the interpretation of the study results and limit the stability and generalizability of the model’s predictive performance (PPIs non‐used, AUC = 0.598 vs. PPIs used, AUC = 0.402, *p* < 0.05) (see Table 3 and Figure 1).

**Table 3:** ROC curve analysis of risk factors for predicting UGIB following DAPT.

Variable	Sensitivity (%)	Specificity (%)	Cutoff value	AUC	95% CI	*p*
G‐17	0.579	0.841	11.225	0.734	0.669–0.800	< 0.001
PRECISE‐DAPT Score	0.857	0.696	14.50	0.794	0.722–0.867	< 0.001
P_2_Y_12_ Receptor Antagonist (Ticagrelor)	0.406	0.747	0.50	0.576	0.498–0.654	0.050
PPIs used (Yes)	0.348	0.457	0.50	0.402	0.524–0.672	0.012
PPIs used (No)	0.652	0.543	0.50	0.598	0.328–0.476	0.012
G‐17 and PRECISE‐DAPT Score	0.739	0.842	0.233	0.844	0.788–0.900	< 0.001
Combined three indicators	0.913	0.653	0.108	0.878	0.831–0.925	< 0.001

*Note:* The analysis was conducted to assess the diagnostic efficacy of individual and combined indicators (G‐17, PRECISE‐DAPT score, P_2_Y_12_ receptor antagonist [ticagrelor]), PPIs used (Yes), PPIs used (No) for upper gastrointestinal bleeding (UGIB) after percutaneous coronary intervention (PCI) with dual antiplatelet therapy (DAPT).

Abbreviations: AUC = area under the curve; CI = confidence interval; cutoff value = optimal threshold for predictive discrimination.

### 3.4. Serum G‐17 Levels, PRECISE‐DAPT Score, and the Relationship With Upper Gastrointestinal Bleeding Following Dual Antiplatelet Therapy

Table 4 results showed that 81.16% (56/69) of patients in the UGIB group had serum G‐17 levels above 11.23 mol/L, which was significantly higher than 43.02% (114/265) in the non‐UGIB group (*χ* ^2^ = 31.87, *p* < 0.001). Additionally, 69.57% (48/69) of patients with UGIB had PRECISE‐DAPT scores over 14.50 points, evidently higher than 14.34% (38/265) in the control group (*χ* ^2^ = 6.29, *p* < 0.001). The comparative differences between the two groups were statistically significant (*p* < 0.001) (Table 4). These findings indicate that the levels of G‐17 higher than 11.23 pmol/L, as well as PRECISE‐DAPT scores beyond 14.50, hold significant exploratory value in identifying patients at high risk of bleeding.

**Table 4:** Relationship between serum G‐17 levels and changes in PRECISE‐DAPT scores in two patient cohorts and upper gastrointestinal bleeding.

Variable	Non‐UGIB group (*n* = 265)	UGIB group (*n* = 69)	*χ* ^2^	*p*
G‐17 (*n* %)			*x* ^2^ = 31.87	< 0.001
≤ 11.23 pmol/L	151 (56.98)	13 (18.84)		
> 11.23 pmol/L	114 (43.02)	56 (81.16)		
PRECISE‐DAPT Score (*n* %)			*x*2 = 6.29	< 0.001
≤ 14.50	227 (85.66)	21 (30.43)		
> 14.50	38 (14.34)	48 (69.57)		

*Note:* Subjects were grouped for comparison using the optimal serum G‐17 level cutoff of 11.23 pmol/L and the precise DAPT score of 14.50 from the ROC curve as thresholds.

Abbreviations: AUC, area under the curve; CI, confidence interval; Cutoff Value, optimal threshold for predictive discrimination.

### 3.5. The Influence of PPIs Use or Non‐Use on the Predictive Value of G‐17 Levels for UGIB

To explore whether PPIs use affects the diagnostic performance of G‐17, the DeLong test was adopted for pairwise AUC comparison. The results revealed that the exploratory performance of G‐17 for UGIB was significantly better in patients not taking PPIs than in those taking PPIs (difference in AUC, 0.332 vs. 0.137, *p* < 0.05), indicating a statistically significant difference (Table 5). The above results suggest that PPI usage is a key factor influencing the predictive performance of G‐17; therefore, patients’ PPIs exposure must be taken fully into account in clinical practice.

**Table 5:** The influence of PPIs use or non‐use on the predictive value of G‐17 levels for UGIB.

Model	Z	*p*	AUC difference	95% CI
G‐17—PPI used	10.094	0.000	0.332	0.268–0.397
G‐17—PPI non‐used	2.380	0.017	0.137	0.024–0.249

*Note:* This study employed the DeLong test to compare the differences in the predictive efficacy of G‐17 for UGIB between the PPIs‐used group and the PPIs‐non‐used group.

Abbreviations: AUC difference, difference in area under the curve; CI, confidence interval; *p*, Probability Value; Z, Standard normal deviate statistic.

### 3.6. The Relationship Between ACS Subtyping, P_2_Y_12_ Receptor Antagonist, and the Occurrence of UGIB Following DAPT

Table 6 presented the findings stratified by ACS subtypes. The proportion of UA cases was 46.4% in the UGIB group versus 74.3% in the non‐UGIB group. The NSTEMI group accounted for 39.1% and 20.0%, respectively, while the STEMI group stood at 14.5% and 5.7% (*χ* ^2^ = 20.31, *p* < 0.001). Multivariate logistic regression showed that the UGIB risk of patients with NSTEMI was 1.288 times that of patients with UA (OR = 1.288, 95% CI: 0.492–3.369). In terms of antiplatelet medication, clopidogrel was used by 59.4% of patients with UGIB and 74.7% of non‐UGIB patients. Ticagrelor application accounted for 40.6% and 25.3% correspondingly (*χ* ^2^ = 6.29, *p* = 0.012). Regression analysis confirmed that ticagrelor use was linked to markedly elevated UGIB risk compared with clopidogrel, with an OR of 15.697 (95% CI: 3.951–62.359, *p* < 0.05). These results imply that NSTEMI, STEMI diagnosis, and ticagrelor treatment can serve as potential indicators for identifying patients at high bleeding risk (Table 6).

**Table 6:** Relationship between ACS classification, P₂Y₁₂ receptor antagonist and the occurrence of UGIB following DAPT therapy.

Variable	Non‐UGIB group (*n* = 265)	UGIB group (*n* = 69)	*χ* ^2^	*p*	OR	95% CI
ACS type (*n* %)			20.31	< 0.001		
UA	197 (74.3)	32 (46.4)			1.000	Reference
NSTEMI	53 (20.0)	27(39.1)			1.288	0.492–3.369
STEMI	15 (5.7)	10 (14.5)			1.065	0.251–4.255
P_2_Y_12_ Receptor Antagonist (*n* %)			6.29	0.012		
Clopidogrel	198 (74.7)	41 (59.4)			1.000	Reference
Ticagrelor	67 (25.3)	28 (40.6)			15.697	3.951–62.359

Note: The study subjects were further grouped according to ACS subtype (NSTEMI, STEMI) and P₂Y₁₂ Receptor Antagonist (clopidogrel, ticagrelor). The relationship between indicators within each group and UGIB was analyzed using *χ* ^2^ tests and multivariate logistic regression.

Abbreviations: CI, confidence interval; OR, odds ratio; *p*, probability value; *χ* ^2^, Chi‐Square.

### 3.7. Comparison of the Incidence of UGIB Between the PPIs+ DAPT Group and the DAPT‐Only Group

Table 7 compared UGIB incidence between the PPI combined DAPT group and the DAPT monotherapy group. The results showed that the incidence of UGIB was significantly lower in the combined group (34.78%) than in the DAPT‐only group (65.22) (*χ* ^2^ = 8.38, *p* = 0.004). This difference was statistically significant (*p* < 0.05) (Table 7). This finding suggests that the addition of PPIs to DAPT treatment may reduce the incidence of UGIB, and may influence the model’s evaluation outcomes for UGIB; therefore, this should be considered carefully.

**Table 7:** Comparison of the incidence of UGIB between the PPI + DAPT group and the DAPT group.

Variables	Non‐UGIB group (*n* = 265)	UGIB group (*n* = 69)	*χ* ^2^	*p*	OR	95% CI
PPIs + DAPT used, *n* (%)	144 (54.34)	24 (34.78)	8.38	0.004	0.007	0.000–0.108
DAPT used, *n* (%)	121 (45.66)	45 (65.22)				

*Note:* The study subjects were further stratified according to whether they were receiving concomitant PPI therapy, divided into a PPI + DAPT group and DAPT group. The *χ* ^2^ test and multivariate logistic regression analysis were used to assess the relationship between the indicators within each group and UGIB.

Abbreviations: CI, confidence interval; DAPT, dual antiplatelet therapy; OR, odds ratio; *p*, probability value; PPIs, proton pump inhibitors; *χ* ^2^, Chi‐Square.

### 3.8. A Comparison of Factors Influencing Early‐ and Late‐Bleeding in Patients With UGIB

Patients were divided into early bleeding (within 30 days after PCI) and late bleeding (30 days post‐PCI to follow‐up endpoint) groups according to the initial onset time of UGIB. Bleeding onset time was set as the dependent variable, and relevant risk factors were defined as independent variables for intergroup comparison. Among all bleeding cases after PCI and DAPT treatment, seven cases belonged to early bleeding and 62 cases to late bleeding; The early bleeding group had markedly higher G‐17 levels (53.02 ± 10.95 pmol/L) than the late bleeding group (32.52 ± 22.35 pmol/L, *p* = 0.001); By contrast, PRECISE‐DAPT scores were obviously lower in early bleeding patients (10.14 ± 8.28) versus late bleeding patients (17.50 ± 7.61, *p* = 0.019). In terms of P2Y12 receptor antagonists, ticagrelor use accounted for 85.71% in the early bleeding group, while clopidogrel was more commonly applied in the late bleeding group at 64.52% (*p* = 0.031). All intergroup differences reached statistical significance (*p* < 0.05) (Table 8). Our findings suggest that elevated G‐17 levels and ticagrelor administration correlate with early UGIB onset. PRECISE‐DAPT score has weak relevance to early bleeding but is closely linked to late bleeding. Further validation is required to confirm these conclusions.

**Table 8:** A comparison of factors influencing early‐ and late‐bleeding in patients with UGIB.

Variables	Early bleeding group (*n* = 7)	Late bleeding group (*n* = 62)	t/Z/*χ* ^2^	*p*
G‐17 (pmol/L)	53.02 ± 10.95	32.52 ± 22.35	*t* = 4.08	0.001
PRECISE‐DAPT score	10.14 ± 8.28	17.50 ± 7.61	*t* = −2.40	0.019
P_2_Y_12_ receptor antagonist (*n* %)			*χ* ^2^ = 4.66	0.031
Clopidogrel	1 (14.29)	40 (64.52)		
Ticagrelor	6 (85.71)	22 (35.48)		
PPIs used (*n* %)			*χ* ^2^ = 0.61	0.434
No	6 (85.71)	39 (62.90)		
Yes	1 (14.29)	23 (37.10)		

*Note:* The study subjects were classified into early and late bleeding groups based on the time of their first UGIB occurrence. The *χ* ^2^ test was used to analyze the relationships between indicators within each group and early bleeding and late bleeding group in patients with UGIB.

Abbreviations: G‐17, gastrin 17; *p*, probability value; PPIs, proton pump inhibitors; *χ* ^2^, Chi‐Square.

## 4. Discussion

In recent years, the incidence of ACS has been rising, posing a serious threat to human health and life [11]. Guidelines recommend that patients diagnosed with ACS should undergo PCI within 12 h of symptom onset (extendable to 24 h for some patients) to restore coronary blood flow. This aims to improve myocardial perfusion, enhance myocardial survival, and reduce the risk of reinfarction [12]. Post‐PCI DAPT constitutes a key strategy for preventing in‐stent thrombosis, reducing events such as MI, stroke, and cardiovascular mortality, and improving long‐term outcomes [13]. However, bleeding (particularly upper gastrointestinal hemorrhage) represents the primary safety concern following DAPT, with bleeding events closely associated with adverse outcomes including increased mortality and prolonged hospital stays [14, 15]. Current research indicates that gastric mucosal injury is significantly more pronounced in patients receiving DAPT compared with those treated with clopidogrel monotherapy, with indications of a higher incidence than those on aspirin monotherapy [16]. Indicators of gastric function, such as abnormal G‐17, which reflects gastric acid secretion disorders, and reduced PGR, suggesting gastric mucosal atrophy, can help quantify the risk of mucosal injury [17, 18]. Therefore, examining the association between these indicators and DAPT‐related bleeding warrants further investigation.

Platelets play a pivotal role in cardiovascular thrombosis, underscoring the importance of antiplatelet therapy, particularly for preventing recurrent atherothrombotic events [19]. Although aspirin and P_2_Y_12_ receptor antagonist inhibit platelet aggregation via distinct mechanisms, aspirin irreversibly inhibits cyclooxygenase‐1 (COX‐1), reducing prostaglandin synthesis and compromising the gastric mucosal barrier. Concurrently, its acidic nature may directly damage gastric mucosal epithelial cells. Current clinical guidelines recommend the use of ticagrelor or prasugrel in preference to clopidogrel for P_2_Y_12_ receptor antagonism. These agents reversibly block P_2_Y_12_ receptors, potently inhibiting platelet aggregation while reducing the release of gastric mucosal protective factors, thereby diminishing mucosal repair capacity. Both may impair gastric mucosal defense mechanisms by exacerbating inflammatory responses [20, 21]. This study used *χ* ^2^ tests and multivariate logistic regression to explore associations of ACS subtypes and P_2_Y_12_ receptor antagonists choice with UGIB. The incidence of UGIB was 40.6% higher in the ticagrelor group, while the incidence of non‐UGIB was 74.7% higher in the clopidogrel group (*p* < 0.05). Multivariate analysis further demonstrated that ticagrelor use was associated with a 15.697‐fold increased risk of UGIB compared with clopidogrel (OR = 15.697, 95% CI: 3.951–62.359). These results confirm that ticagrelor is strongly associated with an increased risk of UGIB. This aligns with prior findings that in elderly patients with ACS undergoing DAPT, both prasugrel and ticagrelor carry increased bleeding risks compared with clopidogrel [22]. However, some studies suggest no significant difference in bleeding risk between ticagrelor and clopidogrel, potentially due to differences in study populations. Such studies may predominantly include younger, healthier individuals. In contrast, this study analyzed the UGIB group, where middle‐aged and elderly patients (aged > 70 years) constituted a higher proportion. This demographic exhibits weaker gastric mucosal defense capabilities, thereby accentuating the bleeding risk differential associated with ticagrelor. This study found that NSTEMI and STEMI were more common in the UGIB group, whereas UA was more frequent in the non‐UGIB group, suggesting a potential link between severe ACS and UGIB. However, after adjustment for P_2_Y_12_ receptor antagonists type, ACS subtype was not independently associated with UGIB. This indicates that ACS subtype may not have a direct causal relationship with UGIB, but reflects clinical practice that critically ill patients with ACS are at higher bleeding risk when receiving potent P_2_Y_12_ receptor antagonists after PCI [23]. Furthermore, clinicians are increasingly inclined to administer potent P_2_Y_12_ receptor antagonists such as ticagrelor following PCI in patients with severe ACS, aiming to reduce the risk of adverse cardiovascular events—including death—among those with more severe subtypes of ACS [24].

Gastrin plays a crucial role in our digestive system by stimulating gastric acid and pepsin secretion, regulating gastrointestinal motility, increasing gastric mucosal blood flow, and promoting gastric mucosal growth and nutrient supply. It is chiefly secreted by G cells in the gastric antrum, body, and duodenum [25]. Gastrin binds to gastrin/cholecystokinin‐B receptors on the cell membranes of parietal cells and enterochromaffin‐like cells, directly elevating intracellular calcium ion concentrations within parietal cells to stimulate gastric acid secretion. This increased acid secretion further stimulates G cells to release G‐17; excessive G‐17 can damage the gastric mucosa, leading to ulcers. Simultaneously, gastric acid activates pepsinogen to form pepsin, which dissolves blood clots and disrupts hemostasis, ultimately leading to upper gastrointestinal ulceration complicated by hemorrhage [18, 26]. The present study demonstrates significantly higher G‐17 levels in the UGIB group compared with the non‐UGIB group. Multivariate logistic regression analysis further shows G‐17 is strongly associated with the occurrence of UGIB following post‐PCI DAPT. This finding may relate to the heightened secretory and stress states of the gastric mucosa in patients with ACS, rendering it more susceptible to DAPT‐induced damage and subsequent upper gastrointestinal hemorrhage. Previous studies have demonstrated that under inflammatory and stress conditions, chief and parietal cells undergo compensatory proliferation, leading to increased secretion of PG. When the gastric mucosa is damaged, PG can permeate the mucosal barrier, resulting in significantly elevated serum levels of PG I and PG II. Activated pepsinogen dissolves blood clots, disrupting hemostatic mechanisms and ultimately causing upper gastrointestinal ulceration with bleeding [27]. This study also found no statistically significant differences in serum PGI, PGII, and PGR levels between the two groups, indicating no clear association between pepsinogen and UGIB following DAPT treatment. This may be partly attributable to factors such as patient diet, smoking, and certain medications (e.g., PPIs). On the other hand, conditions such as gastric mucosal atrophy and dysplasia may exert a more pronounced effect on PG secretion than simple ulcers (whether bleeding or not). This aligns with previous research indicating that elevated serum G‐17 levels correlate with the activity of gastric mucosal lesions, and that higher levels of this marker predict a greater risk of UGIB. In contrast, serum PGI, PGII, and PGR levels lack predictive value [28].

The PRECISE‐DAPT score is a tool used to predict the risk of bleeding in patients undergoing DAPT following PCI. It is constructed based on several clinical indicators, including the patient’s age, creatinine clearance, hemoglobin, white blood cell count, and history of spontaneous bleeding. It quantifies the probability of bleeding by assigning weights to each factor [29]. A large‐scale real‐world study demonstrated that the PRECISE‐DAPT score predicted 5‐year bleeding events (C‐statistic: 0.566, 95% CI: 0.537–0.594), indicating its statistically significant predictive value for long‐term bleeding events in the Chinese PCI population [30]. Furthermore, an international study examining the predictive value of the PRECISE‐DAPT score for bleeding risk in patients undergoing carotid artery stenting (CAS) while on DAPT also demonstrated its statistical significance in predicting post‐DAPT bleeding, with patients scoring ≥ 25 exhibiting a markedly elevated bleeding rate [31]. This study demonstrated that PRECISE‐DAPT score were significantly higher in the UGIB group than in the non‐UGIB group, indicating a strong correlation between this score and the occurrence of UGIB. Stratification by optimal cutoff values of serum G‐17 and PRECISE‐DAPT score further supported the levels of G‐17 higher than 11.23 pmol/L and PRECISE‐DAPT score beyond 14.50 are associated with UGIB in this cohort. Furthermore, the AUC values of G‐17 and PRECISE‐DAPT score alone and in combination were 0.734, 0.794, and 0.844, respectively; when combined with ticagrelor exposure, the AUC further increased to 0.878, indicating that the composite assessment has superior exploratory value in identifying patients at high risk of bleeding (*p* < 0.05). However, this still requires further validation in prospective, multicenter studies.

PPIs potently and persistently inhibit gastric acid secretion by specifically blocking the H^+^‐K^+^‐ATPase enzyme in gastric parietal cells. They are used to treat esophagitis, peptic ulcers, and for the eradication of *Helicobacter pylori*, as well as to prevent ulcers caused by non‐steroidal anti‐inflammatory drugs (NSAIDs) and aspirin [32]. Post‐PCI DAPT is the primary method of preventing thrombotic events in patients with acute myocardial infarction (AMI) who have undergone PCI [13]. However, DAPT significantly increases the risk of UGIB in such patients, and episodes of UGIB may lead to poor outcomes as they can exacerbate underlying heart disease. Not only can bleeding events and the need for transfusions place a significant burden on patients, but the temporary interruption of antiplatelet therapy also increases the risk of ischemic events [14, 15]. The 2018 European Society of Cardiology (ESC) guidelines support the routine use of PPIs in all patients receiving DAPT, whereas the 2010 American College of Cardiology Foundation/American Gastroenterological Association/American Heart Association expert consensus recommends that patients receiving antiplatelet therapy who have other risk factors for bleeding (such as a history of UGIB, advanced age, or co‐administration of warfarin/steroids/NSAIDs/Helicobacter pylori infection) [8]. The results of this study showed that the incidence of UGIB was significantly lower in patients receiving PPIs combined with DAPT compared with those treated with DAPT alone (34.78% vs. 65.22%, *p* = 0.004). Multivariate regression further confirmed that concomitant PPIs use was closely correlated with the risk of UGIB. This implies that the use of PPIs during DAPT may affect the clinical applicability of this model. These findings are highly consistent with previous studies confirming that the addition of PPIs therapy to DAPT significantly reduces the risk of UGIB and all‐cause mortality without adversely affecting major cardiovascular outcomes [33]. Given that PPIs can inhibit gastric acid secretion, thereby promoting the synthesis and release of gastrin, and thus exerting a certain influence on G‐17 levels. The DeLong test was applied to explore the influence of PPIs administration on the association between G‐17 levels and post‐DAPT UGIB. The results revealed that the discriminatory ability of G‐17 for UGIB differed significantly between patients with and without PPIs treatment during DAPT (*p* < 0.05). This indicates that PPIs use may modify the correlation between G‐17 levels and UGIB events, which should be fully considered in clinical practice.

This study also compared patients with early‐ and late‐onset UGIB and found significantly higher G‐17 levels and a higher proportion of ticagrelor use in the early bleeding group, suggesting an association between these two factors and early UGIB. Since the performance of G‐17 may be affected by PPIs administration, the corresponding results should be interpreted with caution. In contrast, the PRECISE‐DAPT score was significantly higher in the late bleeding group, indicating limited value in identifying early bleeding. Nevertheless, our findings remain preliminary and require further prospective multicenter validation before clinical application.

## 5. Study Limitations

We acknowledge several limitations inherent to the present study. First, this was a single‐center retrospective investigation conducted in a cohort characterized by advanced age, a high proportion of NSTEMI/STEMI presentations, and frequent ticagrelor administration. These specific population features limited statistical power and may have compromised the robustness and generalizability of the model, especially for predicting upper gastrointestinal bleeding in younger patients or those with low‐risk acute coronary syndrome. The retrospective, single‐center design also restricts external validity, as the enrolled population may not fully represent broader clinical practice. Consequently, this study merely verified the potential association between the combined predictive model and the risk of UGIB; it cannot confirm its definitive predictive value. To enhance the reliability and generalizability of the research findings, prospective RCTs will need to be conducted in the future. RCTs can control for confounding factors, establish causal relationships, and provide a higher level of evidence.

Second, as this was a retrospective study, bleeding events were identified based solely on clinical presentations, including hematemesis, melena, and signs of systemic hypoperfusion, without endoscopic or other objective confirmatory testing. As a result, minor or self‐limited gastrointestinal bleeding may have been misclassified as clinically relevant DAPT‐related bleeding, potentially leading to overestimated event rates and reduced specificity of the study endpoint. Furthermore, the limitations of medical records mean that some clinical data were incomplete, and several important gastrointestinal confounding factors (such as concomitant use of NSAIDs or anticoagulants, and history of peptic ulcer disease) were not fully incorporated into the analysis. The absence of these variables may result in residual confounding, reduce the accuracy of the model, and lead to an overestimation of the incidence of UGIB and a reduction in the specificity of the endpoint. To address the possible problems of incomplete information or inaccurate records in retrospective studies, future studies should strengthen data quality control and validation measures.

## 6. Conclusion

Higher G‐17 levels and PRECISE‐DAPT scores were associated with UGIB during DAPT after PCI. Combined assessment with ticagrelor exposure showed exploratory value for identifying patients at higher bleeding risk, although PPIs use influenced model performance. Given the retrospective single‐center design and limited sample size, these findings remain preliminary and require prospective multicenter validation.

**Nomenclature**
ACS: acute coronary syndrome
ALT: alanine aminotransferase
AST: aspartate aminotransferase
AUC: area under the ROC curve
BMI: body mass index
BUN: blood urea nitrogen
CREA: creatinine
Cys‐c: cystatin‐C
DAPT: dual antiplatelet therapy
ESC: European Society of Cardiology
G‐17: gastrin 17
GFR: glomerular filtration rate
HB: hemoglobin
HbA1c: glycated hemoglobin
HP: Helicobacter pylori
MI: myocardial infarction
NSTEMI: non‐ST‐segment elevation myocardial infarction
PCI: percutaneous coronary intervention
PG: pepsinogen
PGI: pepsinogens I
PGII: pepsinogens II
PGR: pepsinogen ratio
PLT: platelet
PPIs: proton pump inhibitors
ROC: receiver operating characteristic
STEMI: ST‐segment elevation myocardial infarction
TBA: total bile acid
TBIL: total bilirubin
UA: unstable angina
UGIB: upper gastrointestinal bleeding
WBC: white blood cell
TG: total triglyceride

## Author Contributions

WanPing Yao and Fangfang Li, and Jin Zong were responsible for manuscript writing and funding support. Chang Liu and ZiYi Fang were responsible for data collection and manuscript editing. ShanShan Li was responsible for statistical analysis. Lu Hong Xu and WenHao Qian supervised the findings of this study. All authors discussed the results and contributed to the final manuscript. WanPing Yao and Chang Liu contributed equally to this work.

## Funding

No funding was received for this manuscript.

## Disclosure

All authors approved the final version and agreed to be accountable for all aspects of the work.

## Ethics Statement

The study was conducted in accordance with the Helsinki Declaration and approved by the Ethics Committee of the Affiliated Hospital of Xuzhou Medical University (No: XYFY2025‐KL615‐01). As this was a retrospective study, the ethics committee has waived the requirement for informed consent.

## Consent

The authors have nothing to report.

## Conflicts of Interest

The authors declare no conflicts of interest.

## Data Availability

The data that support the findings of this study are available from the corresponding author upon reasonable request.
